# Quantification of karrikins in smoke water using ultra-high performance liquid chromatography–tandem mass spectrometry

**DOI:** 10.1186/s13007-019-0467-z

**Published:** 2019-07-25

**Authors:** Jakub Hrdlička, Tomáš Gucký, Ondřej Novák, Manoj Kulkarni, Shubhpriya Gupta, Johannes van Staden, Karel Doležal

**Affiliations:** 1 Laboratory of Growth Regulators, Faculty of Science, Institute of Experimental Botany of the Czech Academy of Sciences & Palacký University, Šlechtitelů 27, 78371 Olomouc, Czech Republic; 2 Department of Chemical Biology and Genetics, Centre of Region Haná for Biotechnological and Agricultural Research, Faculty of Science, Palacký University, Šlechtitelů 27, 783 71 Olomouc, Czech Republic; 3 Research Centre for Plant Growth and Development, School of Life Sciences, University of KwaZulu-Natal Pietermaritzburg, Private Bag X01, Scottsville, 3209 South Africa

**Keywords:** Karrikins, Smoke water, Standard dilution method, Standard addition method, Ultra-high performance liquid chromatography (UHPLC), Tandem mass spectrometry (MS/MS)

## Abstract

**Background:**

Karrikins (KARs) are plant growth regulators that promote seed germination and the subsequent growth and development of seedlings of many plant species. In nature they are generated and released by combustion of plant material and promote the restoration of burned ecosystems. Smoke water can be artificially prepared as a saturated extract of all substances in smoke produced by burning plants, and it has various horticultural and agricultural applications.

**Results:**

We have developed, validated and applied the first fast, specific and sensitive method, based on ultra-high performance liquid chromatography–tandem mass spectrometry, for quantifying KARs in smoke water. To assist these efforts and further analyses, standards of the main KARs (which are not commercially available) were synthesized. Due to the complex matrix of smoke waters, two quantification approaches (standard dilution with a structural KAR analogue and standard addition) were compared. The standard addition method allowed absolute quantification of KARs in six of eight smoke water samples of diverse origins and ages.

**Conclusions:**

Our findings reveal differences in both total and relative levels of KARs in smoke water, and indicate that differences in its KAR composition may be linked to variations in its bioactivity.

**Electronic supplementary material:**

The online version of this article (10.1186/s13007-019-0467-z) contains supplementary material, which is available to authorized users.

## Background

Every year, wildfires burn large areas of forests in Australia, South Africa and North America. Following these fires, some species of plants, called fire-followers, rapidly germinate, grow, flower and produce new plants. This is a highly specialised and successful strategy, because these species can rapidly (but temporarily) colonise the open habitats created by fire before competing vegetation re-establishes [1]. Intriguingly, a study published in 1990 showed that smoke derived by burning plants could stimulate seed germination in the same manner as fire [2]. Since then, it has been shown that smoke from burned paper and even extracts prepared from heated agar or cellulose can stimulate germination [3]. Thus, the germination-promoting compound(s) can be produced from commonly occurring plant components. Initially, nitric oxide was considered as the active compound responsible for smoke’s effects on plant growth and development [4].

However, in 2004 the first of a new class of highly active compounds called karrikins (KAR_1_: 3-methyl-*2H*-furo[2,3-c]pyran-2-one) was independently identified in smoke by two groups [5, 6]. The name karrikins, or karrikinolides, is derived from an Aboriginal term for smoke—‘karrik’. The first group studied cellulose-derived smoke (from combustion of filter paper) and elucidated the structure of KAR_1_ from mass and light spectrometric data. Confirmation of the structure was obtained by bioassays with synthetic compounds, and the presence of KAR_1_ in extracts of plant-derived smoke was confirmed by gas chromatography-mass spectrometry (GC–MS) [5]. The second group studied smoke-saturated water derived from burned plants (*Passerina vulgaris* Thoday and *Themeda triandra* L.), identified KAR_1_ by gas chromatography-mass spectrometry (GC-MS), and elucidated its structure by nuclear magnetic resonance (NMR) spectroscopy [6]. They also subsequently produced synthetic compounds [7, 8].

**Fig. 1:**
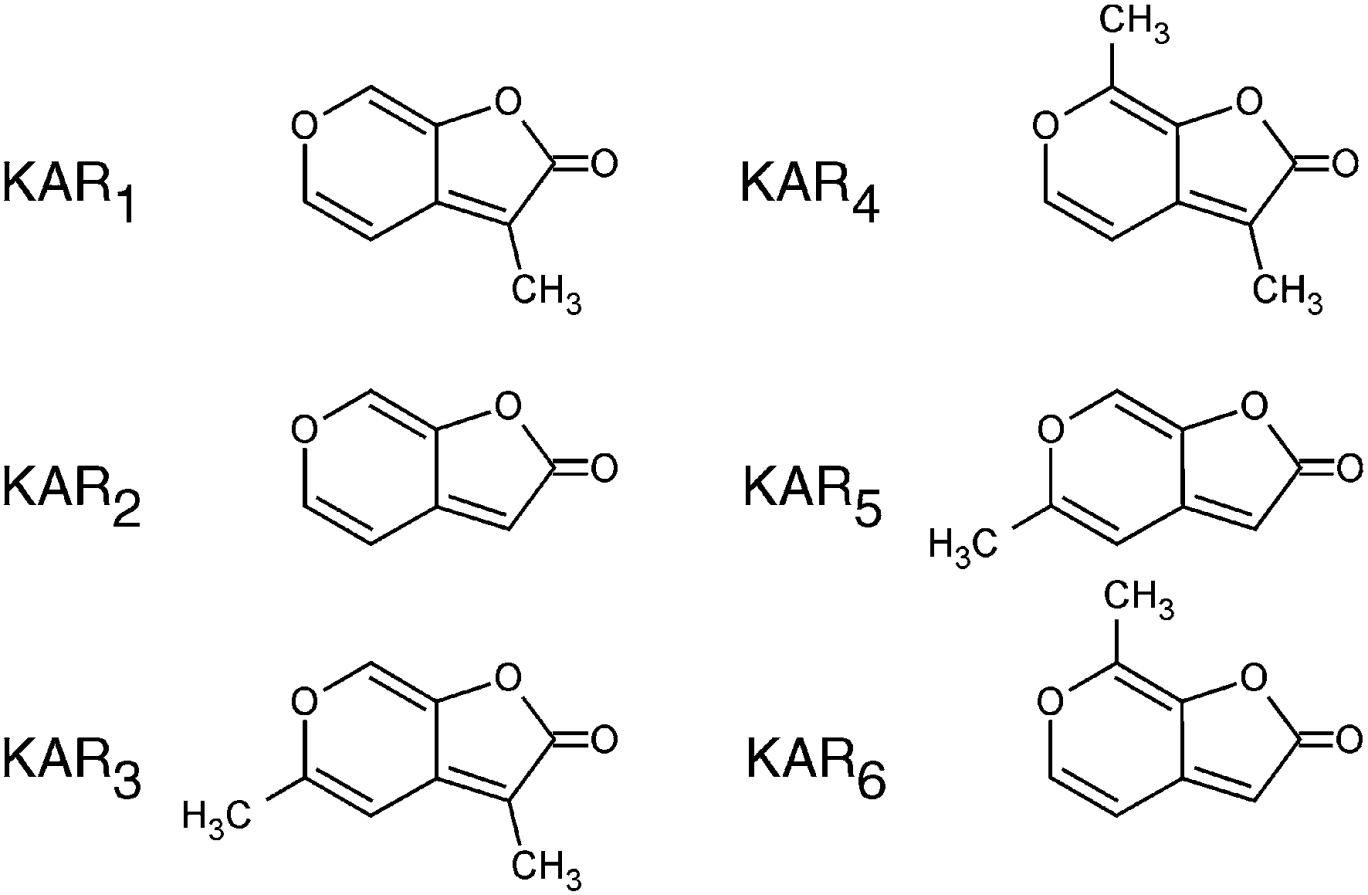
Structures of known karrikin compounds, endogenously occurring in smoke water

A characteristic feature of karrikins is a pyran ring, putatively derived directly from pyranose sugars in plant material, which explains their generation from polysaccharides and amino acids [7]. However, the precise chemical reactions involved in their formation by fire are still unknown. To date, six alkyl analogues of karrikins have been prepared, identified and described (designated KAR_1_ − KAR_6_; Fig. 1) [9, 10]. KAR_1_ does not appear to have mutagenic or genotoxic effects, at least at levels between 10^−10^ and 10^−4^ [11].

KARs are compounds of a class of butenolide derivatives that influence seed germination and both the growth and development of seedlings [1]. The results of germination bioassays with seeds of lettuce (*Lactuca sativa* cv Grand Rapids) and various smoke-responsive species from Australia (*Canostylis aculeate*, *Stylidium affine*), South Africa (*Syncarpha vestita*) and North America (*Nicotiana attenuate*, *Emmenanthe penduliflora*) have shown that the synthetic karrikins have activity at nanomolar concentrations [5, 12].

KARs are not effective on all species of plants [13, 14]. However, to date positive responses to KAR_1_ have been recorded in more than 1200 species, representing 80 phylogenetically diverse plant families. This effect of smoke appears to be independent of plant phylogeny, life cycle, seed structure, ecosystem and geography [15]. Moreover, activity of smoke has been described for non-fire followers, including *Arabidopsis thaliana* and various crop species, such as tomato, maize, rice and lettuce [16–22]. Studies of responses to karrikin in *Arabidopsis* mutants led to the discovery of two essential genes for KARs’ action: *MORE AXILARY GROWTH2* (*MAX2*), previously known to act in the strigolactone signalling pathway, and *KARRIKIN*-*INSENSITIVE2* (*KAI2*), which is similar to the gene encoding the strigolactone receptor DWARF14 (D14) [1, 23]. However, it is still unknown if KARs’ action mode is the same in all plants. Two intriguing questions are how and why non-fire followers have maintained these responses. It has been postulated that KARs may be also created in other natural processes, for example chemical or microbial breakdown of biomass, and/or plant metabolism, and/or they may act due to their similarity to strigolactones [12, 15]. However, none of these hypotheses has been confirmed so far, possibly because of the lack of a sufficiently sensitive, selective and reliable analytical method to detect and quantify endogenous KARs in plant tissues and other biological matrices. An associated bottleneck is a lack of commercially available analytical KAR standards.

KARs are water-soluble substances. Smoke water (SW) is prepared by bubbling smoke generated by controlled combustion of plant material through water. The KARs retain seed germination-promoting activity at very low concentrations, often below 10^−9^ mol/l [8, 15]. Moreover, smoke water tends to have a ‘dual regulatory’ effect on germination, as lower concentrations have a germination-promoting effect, whereas high concentrations of smoke water inhibit germination [24]. 3,4,5-Trimethylfuran-2(5*H*)-one, a compound also recently isolated from plant-derived smoke, is responsible for the germination-inhibiting activity [25]. Therefore, before use the smoke water generally has to be diluted with water in 1:250, 1:500, 1:1000, 1:1500 or 1:2000 (v/v) ratios, depending on the plant species, to maximize its stimulatory biological activity. This relatively easy and economical method provides smoke water that can be stored (after autoclaving) for long periods [6, 26]. An obstacle is that no fast and sensitive method for monitoring karrikins in biological matrices has been previously published, despite the importance of knowing the optimal concentration of KARs in smoke water for both research and practical applications.

To address this hindrance, we have synthesized karrikin standards and developed selective, sensitive analytical protocols for quantifying KARs. We compared two analytical approaches: one based on a standard dilution method (SDM) using 3-bromo-*2H*-furo[2,3-c]pyran-2-one (KAR-Br), a structural analogue of karrikin, as an internal standard (IS), and a standard addition method (SAM). The SAM was successfully validated and applied in the determination of KARs in eight smoke water samples of various origins and ages. The methodology and results are presented and discussed below.

## Results and discussion

### Preparation of karrikin standards

Karrikins are difficult to analyse because they occur in very low concentrations in smoke water, especially in the diluted preparations used in practical applications. Regardless of the methodology used to quantify them, standards of the studied substances would be needed. Thus, as no such substances are commercially available, the first task in this study was to synthesise some.

**Fig. 2:**
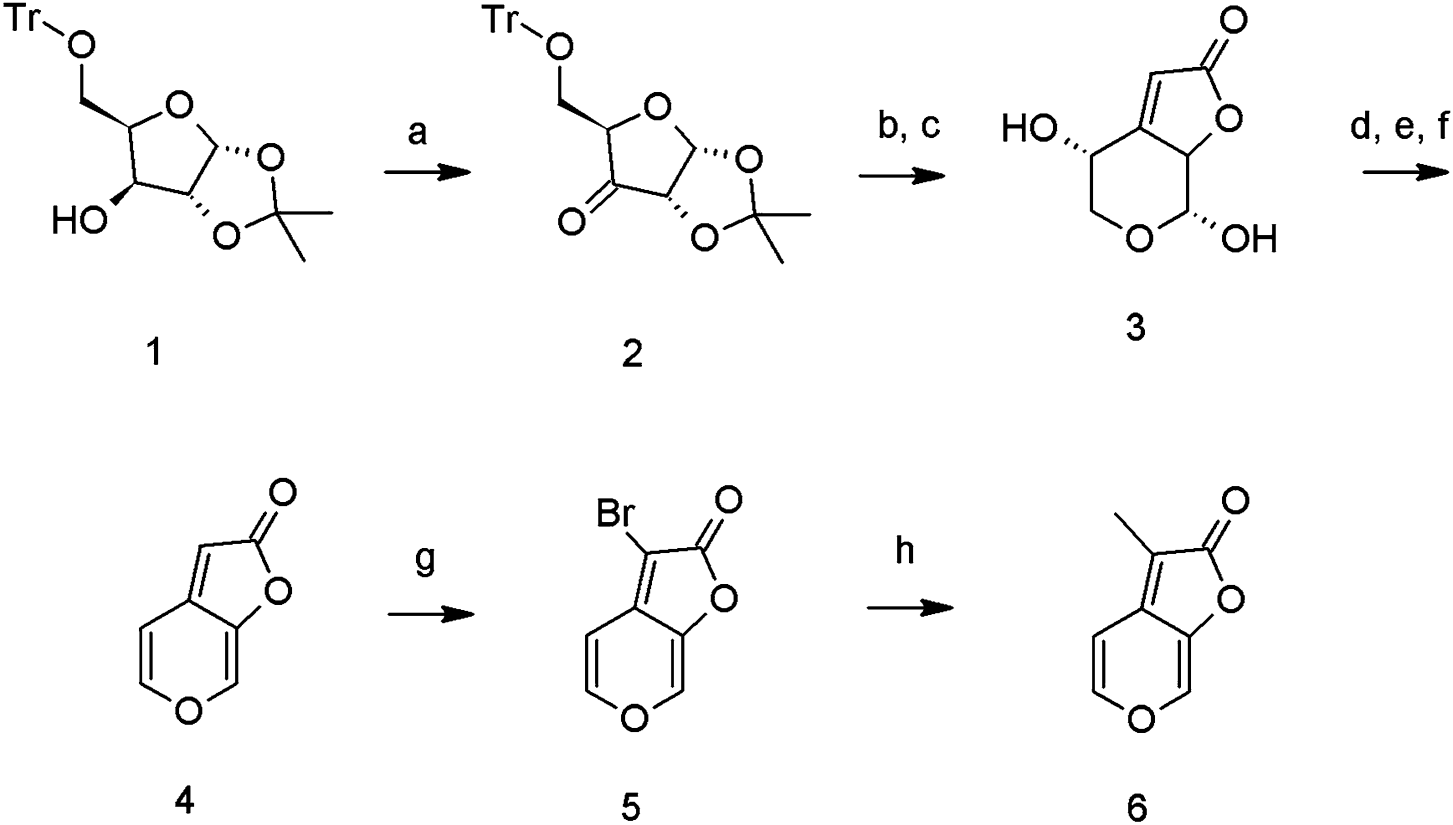
Preparation scheme of karrikins. **a** TEMPO, NCS, TBAB; **b** (EtO)_2_POCH_2_CO_2_Et, NaH; **c** CF_3_COOH; **d** ClCOOEt, pyridine; **e** Et_3_N, CH_2_Cl_2_; **f** Pd(PPh_3_)_4_, THF; **g** NBS, CHCl_3_; **h** CH_3_B(OH)_2_, Pd(OAc)_2_, S-Phos, tBuOK, toluene

The target 3-methyl-*2H*-furo[2,3-c]pyran-2-one (6, KAR_1_) was prepared according to a previously described multistep procedure [27] starting from xylose, with some modification (Fig. 2, Additional file 1). We particularly modified the oxidation of (3a*R*,5*R*,6*S*,6a*R*)-2,2-dimethyl-5-trityloxymethyl-tetrahydro-furo[2,3-d] [1, 3] dioxol-6-ol (1) to (3a*R*,5*R*,6a*S*)-2,2-dimethyl-5-trityloxymethyl-dihydro-furo[2,3-d] [1, 3] dioxol-6-one (2) using TEMPO oxidation. Further stereoselective Horner-Wadsworth-Emmons olefination of ketone (2) with triethylphosphonoacetate was followed by cyclization to (4*R*,7*R*)-4,7-dihydroxy-4,5,7,7a-tetrahydro-furo[2,3-c]pyran-2-one (3) and two-step elimination through bis-ethoxycarbonyloxy intermediate afforded butenolide *2H*-furo[2,3-c]pyran-2-one (4, KAR_2_) in 28 percent overall yield. Position 3- of the furo[2,3-c]pyran-2-one scaffold is susceptible to electrophillic as well as radical substitution. The bromination of (4) proceeded smoothly with excellent yield and purity using *N*-bromosuccinimide in chloroform to 3-bromo-*2H*-furo[2,3-c]pyran-2-one (5, KAR-Br). The bromoderivative (5) was used in a coupling reaction with methylboronic acid, leading to KAR_1_ (6) as previously described [28]. All synthesised standards (KAR_1_, KAR_2_ and KAR-Br) were characterised by ^1^H and ^13^C NMR spectrometry, HPLC–DAD-MS (ESI+) and GC–MS (electron impact, EI) methods (see Additional file 1).

### Stability of karrikin standards

Many secondary metabolites are temperature- and pH-sensitive. They may also lose biological activity through microbial and chemical decomposition during storage [29]. Moreover, they may be unstable during any of the numerous steps in most bioanalytical methods, e.g. collection, dilution, extraction, evaporation and reconstitution of samples and (in mass spectrometric analyses), processes in the ion source [30].

**Fig. 3:**
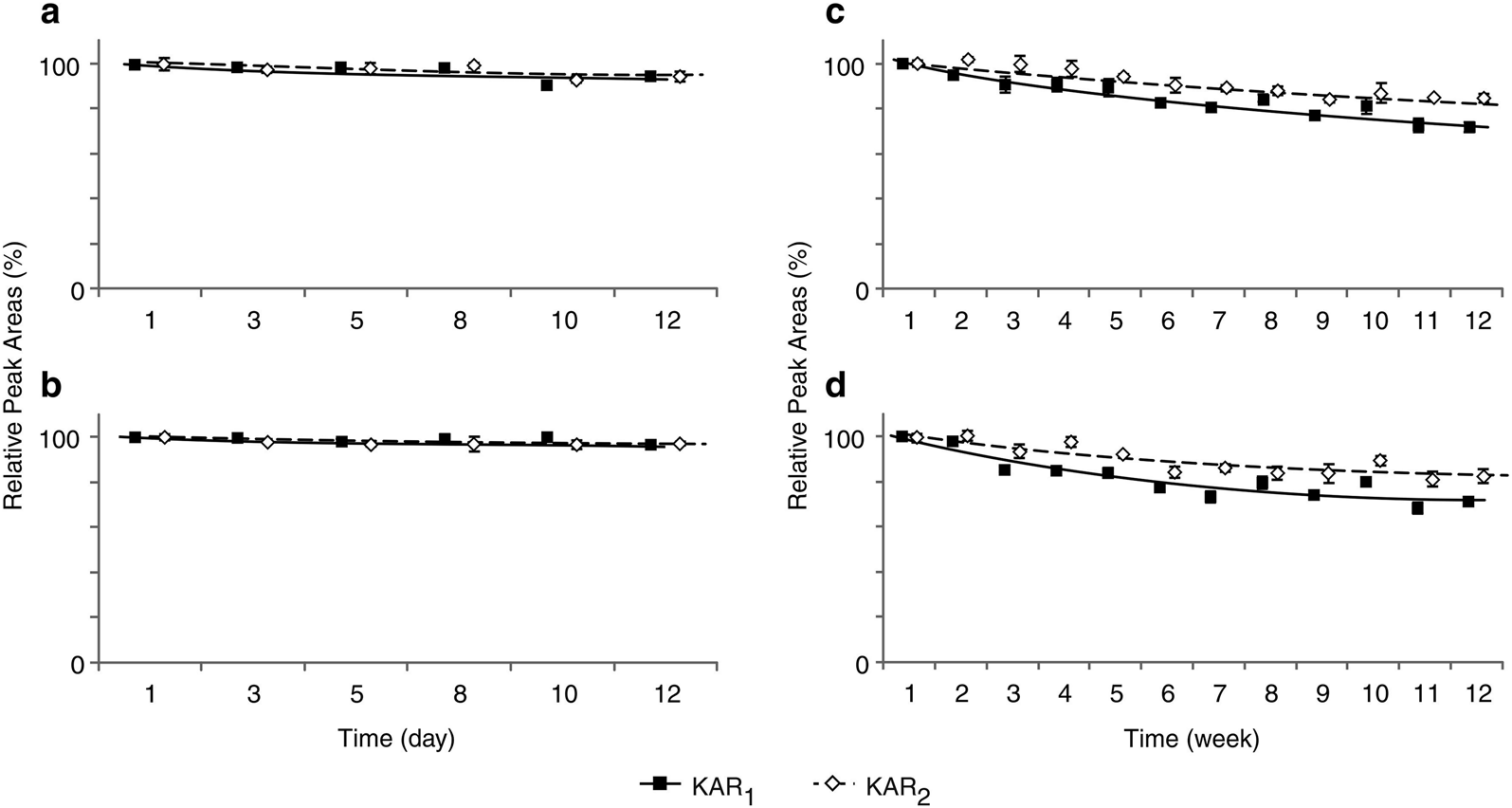
Stability of karrikin standards in McIlvaine buffer, pH 5.0. Solutions of KAR_1_ (black squares) and KAR_2_ (white diamonds) standards (10^−5^ M) were incubated for 12 days (short-time treatment; **a**, **b**) and 12 weeks (long-time treatment; **c**, **d**), at two temperatures: + 22 °C (**a**, **c**) and + 4 °C (**b**, **d**). Values are mean ± SD (n = 3)

Some concentrated smoke extracts, including some stored at 10 °C, have reportedly retained their germination activity in bioassays for many years [31]. We verified that KARs in 10^−5^ mol/l stock solutions did not degrade under our experimental conditions, by analysing stored solutions using a liquid chromatograph coupled to a photo diode array (PDA) and calculating the peak area of each analyte relative to areas of corresponding peaks in reference samples (Fig. 3, Additional file 2). For agricultural and horticultural applications, aqueous solutions of KARs with both neutral and acidic pH have been used [19]. A short-time stability test showed that our KAR_1_, KAR_2_ and KAR-Br standards are sufficiently stable for analysis in both weakly acidic and neutral conditions (pH 5.0 and 7.0, respectively) and at both + 4 and + 22 °C. In short-term (12 days) storage, levels of the tested compounds remained close to those found in fresh control samples, with relative peak areas ranging from 91 to 101% in pH 5.0 McIlvaine buffer (Fig. 3a, b) and from 86 to 103% in deionised water (Additional file 2). The minor losses indicate that degradation of KARs is unlikely to occur in sample processing steps. However, during longer term (12 weeks) storage at pH 5.0 and 7.0, amounts of the KARs retained decreased to 70 and 60%, respectively (Fig. 3c, d; Additional file 2). Interestingly, therefore, the KARs were more stable at the lower pH. The strikingly longer reported retention of biological activity of crude smoke water extracts may be due to differences in pH or the presence of other substances that may stabilize the KARs and/or inhibit their degradation.

### Development of the UHPLC-ESI(+)-MS/MS method

Combinations of tandem mass spectrometry and gas or liquid chromatography are popular analytical techniques for analysis of plant hormones and related compounds, providing the ultrahigh sensitivity and selectivity of mass analysers with excellent separation of analytes in samples with complex biological matrices [32].

Here, karrikins were separated by ultra-fast liquid chromatographic separation, which has become a widely used analytical technique as it provides faster analysis, higher separation efficiency and greater resolution than other analytical techniques [30]. It is also non-destructive, which is advantageous as it enables minimal and straightforward sample preparation without the time-consuming analyte derivatization procedures generally required for GC–MS analyses [33]. However, derivatization may be useful in LC–MS, e.g. in some cases it can improve selectivity by providing helpful fragmentation or enhancing signal to noise ratios by increasing analytes’ masses [34].

**Fig. 4:**
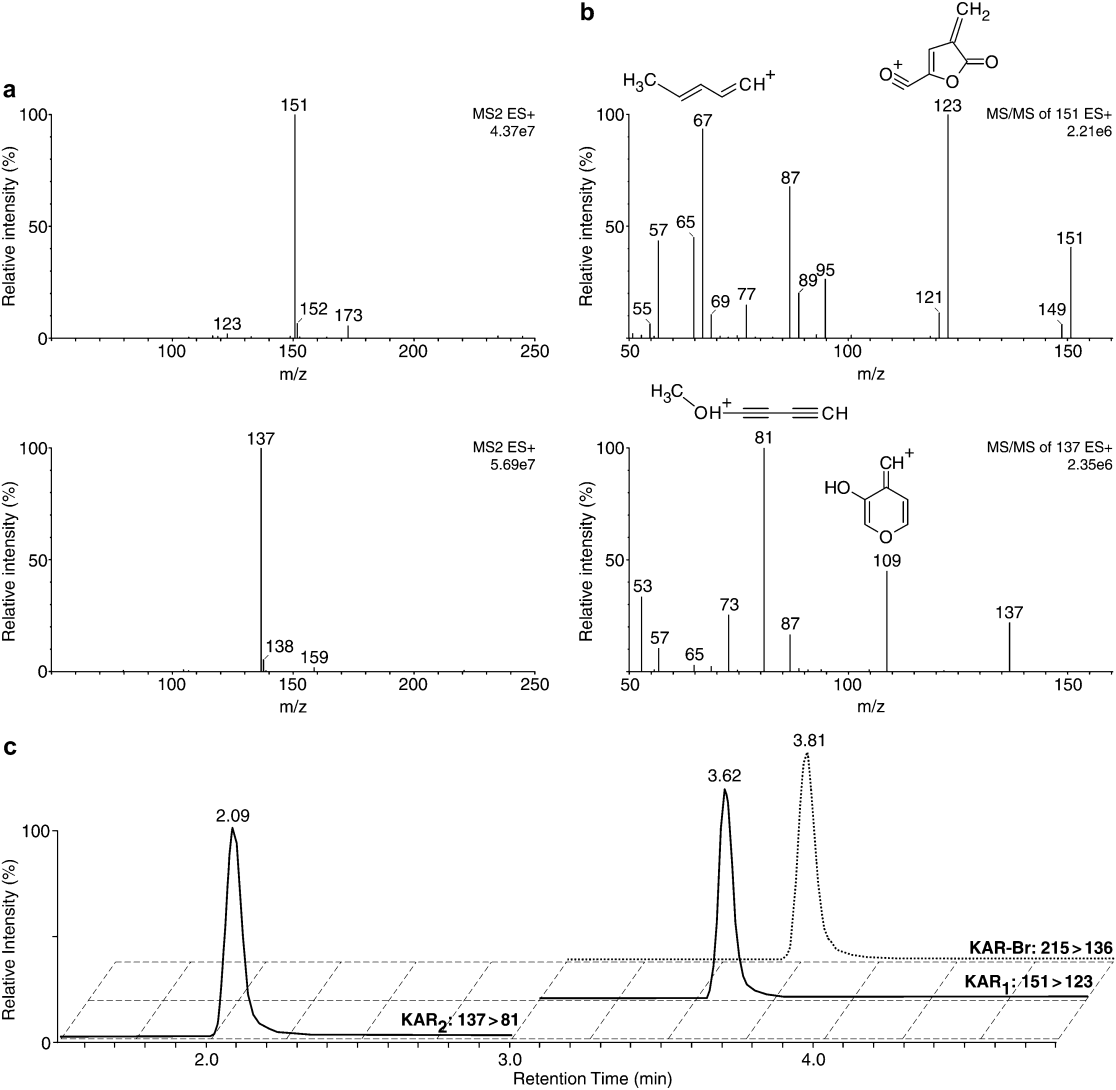
Full-scan (**a**) and product ion (**b**) mass spectra of the studied compounds obtained using the optimised UHPLC-ESI(+)-MS/MS method. MS spectra of KAR_1_ (top) and KAR_2_ (bottom) were analysed with a tandem mass spectrometer Xevo TQ-S in positive ion mode. **c** Separation of KAR standards by the UHPLC-ESI(+)-MS/MS method using an Acquity UPLC BEH C18 2.1 × 50 mm column. Multi-MRM chromatograms of KAR_1_, KAR_2_ and KAR-Br (internal standard) samples, containing 1 pmol of each derivative per injection

More specifically, due to the polar character of karrikinolides (log P < 0; Table 1) and their structural similarity to strigolactones [35], reverse-phase UPHLC was used to separate the studied compounds [36]. The total run time of the chromatographic process, including equilibration, was 7 min. The mobile phase composition and UHPLC gradient were optimised to minimize retention times without compromising peak shapes. Baseline chromatographic separation was achieved using an Acquity UPLC BEH C18 (1.7 µm, 2.1 × 50 mm) column and elution with methanol and water acidified by formic acid (0.1%). A representative UHPLC-MS/MS chromatogram is shown in Fig. 4. The average SD of retention times in a test with 10 consecutive injections of smoke water samples was lower than 0.02 min, confirming that the KAR analytes have high retention time stability under the applied conditions (Table 1).

**Table 1:** Partition coefficients, retention times, diagnostic MRM transitions and optimised instrument settings for the synthesised and studied karrikins

Compounds	log P	R_t_ (min)	CV (V)	Quantitation MRM transitions	CE (eV)	Confirmation MRM transitions	CE (eV)	LOD (fmol)
KAR_1_	− 0.72 ± 0.46	3.62 ± 0.02	35	151 > 123	18	151 > 67	21	1.0
KAR_2_	− 1.31 ± 0.46	2.09 ± 0.01	30	137 > 81	19	137 > 109	16	0.1
KAR-BR	− 0.96 ± 0.50	3.81 ± 0.02	25	215 > 136	22	215 > 80	27	–

*R*_*t*_ retention time, *CV* cone voltage, *CE* collision energy, *LOD* limit of detection

KARs were then detected by a triple quadrupole mass spectrometer equipped with an ESI source in positive ion multiple reaction monitoring (MRM) mode, which has proven utility for simultaneous identification and quantification of plant hormones and related substances [32]. A strong signal was obtained from the singly-charged quasi-molecular ion [M+H]^+^ of each analyte (Fig. 4a). For accurate identification of the major KAR fragments generated by ESI(+)-MS/MS, we used a CFM-ID program predicting the MS/MS fragmentation spectra for a given chemical structure [37]. The most abundant product ion was selected as the quantification transition and the second most abundant as the confirmation transition (Fig. 4b). The key acquisition parameters (cone voltage and collision energy) were optimised for each compound separately. The corresponding retention times, diagnostic MRM transitions and optimised MS/MS conditions used for quantifying and confirming the KARs are listed in Table 1. Based on the reproducibility of the retention times, each chromatographic run was divided into two MRM scan segments (1.5–3.0 and 3.0–5.0 min for KAR_2_ and KAR_1_, respectively), see Fig. 4c. Moreover, the dwell time of each MRM channel was automatically calculated to provide at least 16 scan points per chromatographic peak. The strongest possible signal for each compound was obtained with dwell times ranging from 0.25 to 0.5 s.

Basic validation parameters were calculated after repeatedly injecting solutions with varied concentrations of KAR_1_ and KAR_2_ combined with fixed concentrations of KAR-Br, used as an internal standard. The limit of detection (LOD) and limit of quantification (LOQ) for each analyte were determined from signal-to-noise ratios (3:1 and 10:1, respectively). In the optimised MRM modes, the LODs for KAR_1_ and KAR_2_ were 1.0 and 0.1 fmol, respectively, while their LOQs were close to 2.0 fmol. Linear calibration equations were established from analyses of triplicate samples, in which peak areas for the analytes were compared to those of the IS (KAR-Br). Calibration curves were constructed from peak areas obtained for each analyte at seven concentrations and found to have broad linear ranges, spanning at least four orders of magnitude (1 nmol/l to 10 μmol/l) with coefficients of determination (*R*^*2*^) varying from 0.997 to 0.999.

### Standard dilution method

The standard dilution method (SDM), involving use of an internal standard, is a common method of quantifying analytes in complex biological matrices in MS-based analysis. The most commonly used internal standards are stable isotopically labelled substances, due to the similarity of their chemical properties and retention times to those of non-labelled analogues [38, 39]. A portion of a sample is mixed with a known amount of isotopic standard and the isotopic ratio of the resulting mixture is measured [40]. However, no stable isotope-labelled karrikin standards are available or have been described in the literature. Therefore, a structural analogue, a synthetic brominated derivative of karrikin (3-bromo-furo[2,3-c]pyran-2-one, KAR-Br, see Additional file 1), which is not believed to occur naturally, was used as an internal standard in this study.

To validate the SDM approach, diluted smoke water and deionised water (dH_2_O) were spiked with a mixture of KAR_1_, KAR_2_ (0.5 and 5 μmol/l) and KAR-Br (1 μmol/l). The mixtures were analysed as already described, then concentrations of each analyte calculated from the resulting peak areas were compared with the known amounts added to samples. Levels of the analytes recovered from dH_2_O samples spiked with them at two concentrations were in the range 90% to 110% (Additional file 3). However, yields of KAR_1_ and KAR_2_ in smoke water matrices were, surprisingly, less than 40 and 65%, respectively (Additional file 3). These results were corroborated by the analytical accuracy, expressed as percentage bias, which averaged − 54.3% for KAR_1_ and − 44.7% for KAR_2_. Thus, the SDM’s accuracy did not meet general criteria for bioanalytical methods (nominal thresholds of 15–20%; [41], but it had adequate precision (RSD < 10%) (Table 2). Moreover, peak areas of KAR_1_, KAR_2_ and KAR-Br were substantially decreased, indicating that there were substantial ion-suppressing matrix effects. Thus, it was necessary to test effects of a complex multicomponent smoke water matrix on KAR quantification.

**Table 2:** Validation parameters of standard dilution method and relative matrix effect (ME_Rel_, %) of smoke water samples in karrikin analysis

Compounds	Method precision (% RSD)		Analytical accuracy (% bias)		ME_Rel_ (%)	
	0.5 μmol/l	5 μmol/l	0.5 μmol/l	5 μmol/l	0.5 μmol/l	5 μmol/l
KAR_1_	5.2	8.1	− 65.7	− 42.8	− 79.8	− 61.0
KAR_2_	3.2	7.4	− 68.0	− 21.4	− 71.5	− 53.9
KAR-Br	–	–	–	–	− 28.6	− 34.8

### Matrix effects of smoke water

The mass spectrometric analysis of biologically active compounds in crude plant extracts can be affected by signal-enhancing or -suppressing matrix effects [42, 43], levels of which depend on the analytes’ hydrophobicity and affinity for the stationary phase. Polar analytes (such as karrikins) can be sensitive to ion suppression, while organic solvents generally enhance ESI signals, especially in positive mode [44]. ‘The analytes’ mass and charge can also affect the ion suppression process. Due to competition for available charges, molecules with higher mass will tend to suppress signals from smaller molecules, and analytes’ polarity is generally correlated with their susceptibility to suppression [45]. In our case, reductions in the KAR signal responses observed in the spiked samples of smoke waters led to erroneous results (Table 2; Additional file 3). Therefore, we calculated the relative matrix effects (ME_Rel_) using peak areas obtained for each analyte in the presence and absence of matrix (SW2) to examine the effects in more detail.

The negative value of ME_Rel_ indicates the strength of signal suppression, which can severely impair the reproducibility, linearity and accuracy of analytical methods [43]. We recorded a 30% reduction in peak area of KAR-Br in the presence of matrix, relative to the area in its absence, and corresponding reductions in peak areas of KAR_1_ and KAR_2_ at the two concentrations of 70 and 63%, respectively (Table 2). Thus, the matrix effects were ca. twice as strong for the KAR compounds as for the internal standard (KAR-Br). This difference would severely affect the method’s accuracy and highlights the importance of using stable isotope-labelled internal standards to ensure that signals from analytes and internal standards are virtually identical. However, no stable isotope-labelled KAR standards are currently available, so the matrix effects in the SDM could not be eliminated and we applied calibration (the standard addition method) to account for them instead.

### Standard addition method

The standard addition method (SAM) is an analytical approach that involves spiking samples with known amounts of appropriate standards and plotting the resulting peak areas of analytes against the concentration of added standards. Within linear response ranges this should provide a straight line, *y *=* kx *+* q*, where *x* is analyte concentration, *y* is area, *q* is the intercept and *k* the slope [46]. SAM is a very reliable method, but sample preparation, measurement and calculation are time consuming [47].

**Fig. 5:**
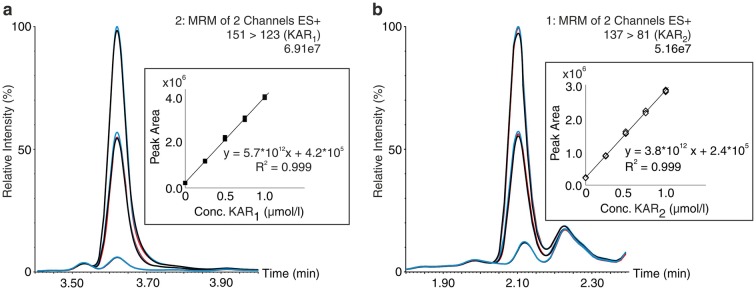
Representative MRM chromatograms of SW5 samples, non-spiked and spiked with a mixture of KAR_1_ (**a**) and KAR_2_ (**b**) at two concentrations (0.5 and 1 μmol/l). Reproducibility is shown in triplicates (1st injection blue, second injection red, third injection black). In the boxes, representative calibration curves constructed from five SW matrix-based solutions spiked with a mixture of KAR_1_ and KAR_2_ at known concentrations (0, 0.25, 0.5, 0.75 and 1.0 μmol/l). The samples were analysed by UHPLC-ESI(+)-MS/MS using the standard addition method

For each smoke water sample, calibration curves were constructed using five matrix-based solutions spiked with a mixture of KAR_1_ and KAR_2_ at known concentrations (0, 0.25, 0.5, 0.75 and 1.0 μmol/l). Representative MRM chromatograms of one of the spiked smoke water samples (designated SW5) and calibration curves for KAR_1_ and KAR_2_ in it are shown in Fig. 5. Across a working linear range between 0 and 1.0 μmol/l, the coefficients of determination (*R*^*2*^) ranged from 0.984 to 0.999 (Table 3). Overall, our results showed that the methods has good linearity, so levels of KAR_1_ and KAR_2_ in the smoke water samples were finally calculated by linear regression (Table 3).

**Table 3:** Levels of karrikins (KAR_1_ and KAR_2_) determined in smoke water samples using the standard addition method

Samples	Calibration curve (KAR_1_)	R^2^	KAR_1_ (nmol/l)	Calibration curve (KAR_2_)	R^2^	KAR_2_ (nmol/l)	KAR_1_:KAR_2_ ratio
SW1_1993	y = 9.42*10^12^ (± 1.2*10^11^)x + 1.1*10^5^ (± 4.2*10^4^)	0.997	11.7 ± 4.3	y = 3.2*10^12^ (± 1.2*10^11^)x + 6.9*10^4^ (± 1.1*10^4^)	0.992	21.7 ± 2.7	0.5
SW2_1998	y = 9.1*10^12^ (± 1.0*10^11^)x + 3.0*10^5^ (± 1.0*10^4^)	0.999	32.5 ± 1.4	y = 5.3*10^12^ (± 7.0*10^10^)x + 1.5*10^5^ (± 1.9*10^4^)	0.998	28.9 ± 4.1	1.1
SW3_1994	y = 3.9*10^12^ (± 4.1*10^10^)x + 2.5*10^5^ (± 5.5*10^3^)	0.999	63.2 ± 1.5	y = 2.6*10^12^ (± 2.7*10^10^)x + 2.3*10^5^ (± 3.8*10^3^)	0.999	88.7 ± 1.4	0.7
SW4_1999	y = 1.1*10^13^ (± 5.7*10^10^)x + 1.1*10^5^ (± 4.5*10^3^)	0.998	9.4 ± 0.4	y = 7.0*10^12^ (± 3.6*10^10^)x + 10.0*10^4^ (± 1.1*10^4^)	0.999	14.3 ± 1.5	0.7
SW5_2003	y = 5.7*10^12^ (± 4.9*10^10^)x + 4.2*10^5^ (± 1.8*10^4^)	0.999	74.4 ± 3.7	y = 3.8*10^12^ (± 4.2*10^10^)x + 2.4*10^5^ (± 1.1*10^4^)	0.999	64.2 ± 3.5	1.2
SW6_2003	y = 3.9*10^12^ (± 6.2*10^10^)x + 3.7*10^5^ (± 1.4*10^4^)	0.999	101.6 ± 7.9	y = 2.5*10^12^ (± 3.6*10^10^)x + 7.1*10^4^ (± 2.2*10^3^)	0.999	28.8 ± 1.7	3.5
SW7_2003	y = 1.0*10^13^ (± 5.5*10^10^)x + 1.6*10^6^ (± 5.0*10^4^)	0.984	nd	y = 9.5*10^12^ (± 1.4*10^11^)x + 6.9*10^5^ (± 7.9*10^4^)	0.998	nd	–
SW8_2004	y = 1.0*10^13^ (± 1.6*10^11^)x + 1.7*10^6^ (± 5.3*10^4^)	0.990	nd	y = 9.5*10^12^ (± 1.3*10^11^)x + 8.8*10^5^ (± 5.2*10^4^)	0.999	nd	–

### Quantification of karrikins in smoke water

Understanding the optimal concentration of smoke water to apply to smoke-responsive seeds is important, as some concentrations may be toxic while others promote germination, depending on the levels (absolute and relative) of inhibitory or stimulatory compounds present. The preparation method, storage conditions and storage period may also affect its activities. Thus, we analysed concentrations of the germination-stimulating molecules KAR_1_ and KAR_2_ in smoke extracts prepared from various plant materials, or purchased commercially, and stored for different periods (as described in *Materials and methods*). Smoke extracts prepared from fynbos vegetation, *Themeda triandra* grass, and leaf material from montane grassland species all reportedly have germination-promoting effects on *T. triandra* seeds [48, 49]. In addition, extracts of smoke obtained from various plant materials and even laboratory tissue paper have stimulated germination of light-sensitive Grand Rapids lettuce seeds in the dark [3]. These findings indicate that generally all types of plant material can be used to prepare smoke extracts, as already stated.

Using the validated UHPLC-ESI(+)-MS/MS method, levels of KARs in different types of smoke water were quantified by standard addition measurements, and the results confirmed (Table 3) that they had varying levels of KAR_1_ and KAR_2_. The lowest level of KAR_1_ was detected in the oldest extract (SW1), and its KAR_2_ content was twice as high. SW of two ages obtained from burning *T. triandra* (SW3 and SW4) had the same KAR_1_ to KAR_2_ ratio (and higher levels of KAR_2_ than KAR_1_), but different absolute levels. The highest level of KAR_1_ and largest difference between KAR_1_ and KAR_2_, was detected in a factory concentrate (SW6). In both commercial extracts examined (SW7 and SW8) no KARs were detected. Nelson et al. [15] reported that KAR_1_ is abundant in smoke, and it is believed to be the most abundant and active karrikin derivative. However, our results show that KAR_2_ is more abundant in some smoke waters. We cannot tell as yet whether these differences are due to differences in tested smoke waters’ age or origin. Additionally, we did not detect any KARs in pure water samples (Additional file 5: Fig. S2).

## Conclusions

To our knowledge, no methodology suitable for monitoring KARs in biological matrices has been previously published. Thus, in the presented study we developed a UHPLC-ESI(+)-MS/MS method for analysing karrikins in different types of smoke water. The method was then successfully applied to analyse KARs in smoke water of various origins. In the future, this new approach should be useful for studying karrikins’ physiological effects on plant growth, development and metabolism, as well as their modes of action in plants or soil. The described method is functional and robust, but due to the complexity of smoke water matrices, isotope-labelled standards will be required for routine analyses.

## Methods

### Chemicals and reagents

Methanol (gradient grade for liquid chromatography) was obtained from Merck (Darmstadt, Germany) and formic acid from Sigma-Aldrich (St. Louis, MO, USA). Water was purified using a Simplicity™ System (Millipore, Bedford, MA, USA). The reagents for chemical synthesis were purchased from Sigma-Aldrich.

### Biological materials

Bioactive molecules present in smoke can be much more conveniently applied to plants in smoke water than in smoke aerosols [31]. There are several available methods for preparing smoke water, but they are still crude and need refinement. In a common approach, smoke is generated by burning plant materials [50] in aerated drums and bubbled in compressed air through water [2]. Diverse plant materials are suitable for preparing smoke extracts, but not latex-containing material [3]. The smoke water extract (SW) samples used in this study were all prepared (in-house or purchased for research work) in this manner. For karrikin analysis we used six types of extracts prepared in-house from: 5 kg fynbos vegetation in 1993 (SW1); 5 kg *Passerina vulgaris* and *T. triandra* in 1998 (SW2), 5 kg *T. triandra* grass in 1994 (SW3), 10 g of *T. triandra* grass used in a pilot apparatus, in 1999 (SW4); 5 kg fynbos vegetation in 2003 (SW5). We also used samples of three commercial products: smoke distillate factory concentrate, prepared in 2003 (SW6), Fire Grow™ (SW7) and Super smoke plus (2004, SW8). These samples were stored at 10 °C and diluted 10-fold before analysis. The protocol for preparing smoke–water samples is explained using flow chart in Additional file 4: Fig. S1. Additionally, we analysed also KARs in pure water samples (Additional file 5: Fig. S2).

### Synthesis of karrikin standards

(3a*R*,5*R*,6*S*,6a*R*)-2,2-dimethyl-5-trityloxymethyl-tetrahydro-furo[2,3-d] [1, 3] dioxol-6-ol (1) was prepared according to a previously described procedure with minor modifications [51]. The preparation of target compounds (4), (5) and (6) is described in detail in Additional file 1. The identity and purity of prepared compounds were checked by ^1^H and ^13^C NMR spectrometry, HPLC–DAD-MS (ESI +) and GC–MS (EI) methods.

^1^H and ^13^C-NMR spectra were recorded using a Jeol 500 ECA instrument operating at 500 MHz for ^1^H and 126 MHz for ^13^C, respectively. Chemical shifts are reported in ppm. Coupling constants (*J*) are reported in Hertz (Hz), and the following abbreviations are used: singlet (s), doublet (d), triplet (t), multiplet (m). Mass spectra were recorded using an LCQ ion trap mass spectrometer (Finnigan MAT, San Jose, CA, USA). The purity of the compounds was determined by HPLC–DAD-MS, using an Alliance 2695 separations module (Waters) linked to a PDA 996 (Waters) and Q-Tof micro (Waters) benchtop quadrupole orthogonal acceleration time-of-flight tandem mass spectrometer. Samples of the analytes were dissolved in methanol and diluted to 10 μg ml^−1^ in the initial mobile phase. Then, 10 μl portions of the solution were injected onto a Symmetry C18 RP-column (150 mm × 2.1 mm, 3.5 μm; Waters) housed in a thermostat at 25 °C. The analytes were eluted with a 24-min binary linear gradient of 10 to 90% methanol, balanced with 15 mM formic acid adjusted to pH 4.0 with ammonium hydroxide, at a flow rate of 0.2 ml min^−1^. The gradient was followed by a 10 min isocratic wash with 90% methanol, and 10 min re-equilibration to the initial conditions. The effluent was introduced into the DAD (scanning range 210–400 nm, 1.2 nm resolution) and an electrospray source (source temperature 110 °C, capillary voltage + 3.0 kV, cone voltage + 20 V, desolvation temperature 250 °C). Nitrogen was used as both the desolvation gas (500 l h^−1^) and cone gas (50 l h^−1^). The mass spectrometer was operated in positive (ESI+) ionization mode, and data were acquired in the 50–1000 m/z range. Merck Kieselgel 60 (230–400 mesh) silica gel was used for column chromatography. The purity of biologically evaluated compounds was > 98% according to HPLC–DAD–MS and elemental analysis. GC–MS analyses were performed using a QP2010 Ultra GC–MS instrument (Shimadzu) equipped with a Zebron ZB-5MS capillary column (30 m long, 0.32 mm inner diameter, 0.25 µm film). Helium was used as carrier gas with a constant flow of 1.20 ml min^−1^, and 1 µl portions of samples with 1.0 µg mL^−1^ of analyte were injected in splitless mode The injector temperature was 260 °C, sampling time 1 min, and solvent cut time 1.5 min. The temperature programme consisted of 60 °C held for 1 min, followed by a 20 °C min^−1^ rise to 280 °C, which was held for 5 min. The interface temperature was 280 °C. The ion source was operated with 70 eV collision energy at 250 °C and detector voltage 0.7 kV. Mass spectra were acquired from 50 to 650 m/z scans at 2000 scans·s^−1^.

### Karrikin stability

The stability of KAR_1_, *2H*-furo[2,3-c]pyran-2-one (KAR_2_) and KAR-Br at pH 5.0 and 7.0 was evaluated by preparing solutions of 10^−3^ M standards in methanol and diluting to 10^−5^ M in McIlvaine buffer, pH 5.0 [52], or deionised water (dH_2_O). 1 μl portions of these solutions were injected into an Acquity UPLC^®^ H-Class System equipped with an Acquity UPLC^®^ BEH C18 reversed-phase column (1.7 µm, 2.1x50 mm) and eluted using the binary linear gradient described below, immediately and after 12 days incubation at 4 and 22 °C. Analytes were detected (at 330 nm) with an Acquity PDA detector operating with a scanning range 190–400 nm and 1.2 nm resolution. The analyses were repeated three times, and the entire experiment was repeated with a 21-week incubation using fresh stocks of KAR standards. Relative peak areas of the analytes at the end of the incubations were calculated as percentages of the corresponding peak areas obtained for reference samples at the beginning of the experiment.

### UHPLC-ESI(+)-MS/MS conditions

For quantitative analysis of karrikins, ultra-high performance liquid chromatography-electrospray positive ionization tandem mass spectrometry (UHPLC-ESI(+)-MS/MS) was employed, using an Acquity UPLC^®^ I-Class System (Waters, Milford, MA, USA) combined with a Xevo™ TQ-S triple quadrupole mass spectrometer (Waters, Manchester, UK). Acquired data were processed by MassLynx™ MS Software with TargetLynx™ (version 4.1, Waters, Milford, MA, USA). Portions (2 μl) of diluted samples were injected onto an Acquity UPLC^®^ BEH C18 reversed-phase column (1.7 µm, 2.1 × 50 mm). The analytes were eluted with a 5-min gradient consisting of 5% solvent A (0.1% formic acid in methanol) and 95% solvent B (0.1% formic acid in water) for 0–1 min, followed by a 5–20% linear increase in A from 1 to 3 min, followed by 20% A from 3 to 5 min. The column was then washed with 100% methanol for 1.0 min, and re-equilibrated to initial conditions (1 min, 5% A). The flow rate was 0.4 ml min^−1^ throughout, the column thermostat was set at 40 °C and the autosampler was precooled to 4 °C. Optimised instrument settings were as follows: capillary voltage, + 0.5 kV; source temperature, 150 °C; desolvation temperature, 600 °C; LM/HM resolution 3.0/15.0; ion energy 1/2 0.7/0.1 V; entrance/exit voltages 0.5 V. Nitrogen was used as desolvation gas (550 l h^−1^), cone gas (150 l h^−1^) and nebulizer gas (7 bar). Argon was used as collision gas with an optimised flow of 0.15 ml min^−1^. The MS instrument was operated in multiple reaction monitoring (MRM) mode, monitoring the quasi-molecular ion [M+H]^+^, as the precursor ion and an appropriate product ion. The MS/MS parameters including dwell time (automatic mode for 16 scan points per peak), cone voltage (25–35 V) and collision energy (16–27 eV) were selected to maximize the sensitivity of exact diagnostic transitions. All optimised MS settings of the instruments for individual compounds are listed in Table 1.

### Test of matrix effects

Matrix effects (ME) were assessed using the standard mixture of KARs at two concentrations (0.5 and 5 μmol/l) added to the smoke water SW2 diluted 10-fold and pure dH_2_O (as reference). All samples were prepared in quadruplicate and analysed by the UHPLC-ESI(+)-MS/MS method described above. The strength of the ME was calculated using the post-extraction addition approach [53, 54], in terms of the peak area of analytes in spiked samples of diluted smoke water divided by peak areas of corresponding analytes in standard solution, multiplied by 100. Relative matrix effects (ME_Rel_) were subsequently calculated as ME_Rel_ = ME − 100. A positive value of ME_Rel_ indicates signal enhancement, while negative values indicate signal suppression [55].

### Karrikin quantification

Due to the lack of available KAR analogues labelled with a radioactive or stable isotope, the standard dilution method (SDM) was used for quantification, with KAR-Br (3-bromo-*2H*-furo[2,3-c]pyran-2-one) as an internal standard (IS). Calibration curves were created from KAR standards by plotting known concentrations of each unlabelled analyte, ranging from 0.1 nmol/l to 100 μmol/l, and a defined, fixed concentration of the corresponding IS (1 μmol/l). The ratio of unlabelled standards to IS was used to estimate the concentration of endogenous compounds in the samples. To test the precision and accuracy of the SDM, non-spiked and spiked samples (the latter with 0.5 and 5 μmol/l of KAR_1_ and KAR_2_) of 10-fold diluted smoke water were used. KAR-Br was added as an internal standard at a total concentration of 1 μmol/l. Portions (2 μl) of the samples were analysed by UHPLC-ESI(+)-MS/MS. The final concentrations of added KAR standards were calculated after subtracting their determined endogenous levels, obtained from non-spiked samples. Finally, determined analyte concentrations were compared with known theoretical amounts of appropriate standards added to samples and presented as method accuracy (expressed as percentage bias). The method precision for each analyte was calculated as the relative standard deviation (% RSD) of its determined concentration in four replicates of spiked samples.

Subsequently, KAR standards and smoke water extract matrices were used during development of the standard addition method (SAM). Calibration curves were constructed by adding KAR_1_ and KAR_2_ standards at known concentrations (0, 0.25, 0.5, 0.75 and 1 μmol/l) in three independent repetitions to the smoke water, then analysed by UHPLC-ESI–MS/MS. Levels of KAR_1_ and KAR_2_, present in samples of non-spiked smoke water were subsequently calculated using regression equations.

## Additional files

**Additional file 1.** Preparation and characterization of karrikin compounds.

**Additional file 2.** Results of test of stability of karrikin standards in deionised water, pH 7.0. Solutions of KAR_1_ (black squares) and KAR_2_ (white diamonds) standards (10^−5^ M) were incubated for 12 days (short-term treatment; a-b) and 12 weeks (long-term treatment; c-d) at +22 °C (a, c) and +4 °C (b, d). Values are mean ± SD (n = 3).

**Additional file 3.** Karrikin levels (μmol/l) determined by the standard dilution method. Diluted smoke water (SW) and deionised water (dH_2_O) were spiked with mixtures of KAR_1_, KAR_2_ (0.5 and 5 μmol/l) and KAR-Br (1 μmol/l) then analysed by the presented UHPLC–ESI(+)-MS/MS method. The calculated concentrations of each analyte were compared with the known amounts added to samples – 0.5 μmol/l (a) and 5 μmol/l (b), and the recoveries (%) obtained in each spiking experiment are shown (mean ± SD, n = 4).

**Additional file 4: Fig. S1.** The detailed protocol of smoke–water samples.

**Additional file 5: Fig. S2.** Representative MRM chromatograms of pure water samples (tap water injection blue, distilled water injection red, STD injection black). The samples were analysed in triplicate by UHPLC–ESI(+)-MS/MS using the standard addition method.

## Data Availability

The datasets used and/or analysed during this study are available from the corresponding author on reasonable request.

## Abbreviations

KAR: karrikin
SW: smoke water
dH_2_O: deionised water
IS: internal standard
SDM: standard dilution method
SAM: standard addition method
GC–MS: gas chromatography–mass spectrometry
UHPLC: ultra-high performance liquid chromatography
MS/MS: tandem mass spectrometry
MRM: multiple reaction monitoring
ESI(+): electrospray ionization in positive mode
EI: electron impact
LOD: limit of detection
LOQ: limit of quantification
*R*^*2*^: coefficient of determination
ME: matrix effect
RSD: relative standard deviation
