# Supplementary material for: Quantification of karrikins in smoke water using ultra-high performance liquid chromatography–tandem mass spectrometry
Source: Plant Methods. 2019 Jul 25;15:81. doi: 10.1186/s13007-019-0467-z (PMC6659305; doi:10.1186/s13007-019-0467-z)
Supplement: Supplementary file 1 — Additional file 1. Preparation and characterization of karrikin compounds. [file 13007_2019_467_MOESM1_ESM.docx]

**Additional file 1.** Preparation and characterization of karrikin compounds.

**Preparation of (3a*R*,5*R*,6a*S*)-2,2-dimethyl-5-trityloxymethyl-dihydro-furo[2,3-d][1,3]dioxol-6-one (2)**

To a suspension of *N*-chlorosuccinimide (30.5 mmol), (2,2,6,6-tetramethyl-piperidin-1-yl)oxyl (2.64 mmol) and tetrabutylammonium bromide (2.75 mmol) in a mixture of 0.5 M sodium hydrogencarbonate (55 mL) and 0.5 M potassium carbonate (55 mL) a solution of (3a*R*,5*R*,6*S*,6a*R*)-2,2-dimethyl-5-trityloxymethyl-tetrahydro-furo[2,3-d][1,3]dioxol-6-ol (25.4 mmol) in dichloromethane (110 mL) was added dropwise at room temperature over 30 minutes. The mixture was stirred at room temperature for a further 3 hours. The organic layer was separated and the water phase was extracted with dichloromethane. The combined organic phases were washed with brine, dried over sodium sulphate and evaporated under reduced pressure. The crude product was crystallized from petroleum ether.

Yield: 85 %. Elemental analysis: Calcd.for C_27_H_26_O_5_ (430.49): C, 75.33; H, 6.09. Found: C, 75.01; H, 6.25. HPLC-DAD purity: 98.9 %. HPLC-MS (ESI+): 431.52 (100.0%).

^1^H NMR (CDCl_3_): 1.49 (s, 6H, 2xCH_3_), 3.33 (dd, J=10.08 Hz, J´=2.49 Hz, 1H, CH), 3.52 (dd, J=10.08 Hz, J´=2.49 Hz, 1H, CH), 4.43 (t, J=1.55 Hz, 1H, CH), 4.56 (d, J=7.52 Hz, 1H, CH), 6.35 (d, J=7.52 Hz, 1H, CH), 7.26-7.39 (m, 15H, ArH).

**Preparation of (4*S*,7*S*)-4,7-dihydroxy-4,5,7,7a-tetrahydro-furo[2,3-c]pyran-2-one (3)**

**Step A:**

Triethylphosphonoacetate (24.52 g, 109.2 mmol) was added dropwise to a suspension of sodium hydride oil suspension (55-60 % sodium hydride content) (4.48 g) in dry tetrahydrofuran (200 mL) at -15 °C. The mixture was stirred for further 10 minutes at -15 °C and a solution of compound (2) (23.44 g, 54.6 mmol) in dry tetrahydrofuran (200 mL) was added dropwise over 20 minutes at -15 °C. The mixture was concentrated under reduced pressure. The residue was poured with saturated ammonium chloride solution (200 mL) and extracted twice with ethylacetate. Combined organic phases were washed with water and brine, dried over sodium sulphate and evaporated under reduced pressure. The crude product (25.7 g, yield 94%) was used without purification in the next step.

**Step B:**

The [(3a*R*,5*S*,6a*R*)-2,2-dimethyl-5-trityloxymethyl-dihydro-furo[2,3-d][1,3]dioxol-(6*Z*)-ylidene]-acetic acid ethyl ester (20.0 g, 40.0 mmol) from the previous step was dissolved in dichloromethane (25 ml) and the solution was cooled to 0 °C. A mixture of trifluoroacetic acid (27.0 g, 237 mmol) and water (4.55 g, 253 mmol) was added dropwise with stirring so the temperature was maintained between 0 – 5 °C. After addition the reaction mixture was warmed to room temperature and stirred for a further 5 minutes. The mixture was evaporated under reduced pressure and the crude product was crystallized from ethanol to obtain (4*S*,7*S*)-4,7-dihydroxy-4,5,7,7a-tetrahydro-furo[2,3-c]pyran-2-one (3) as a white solid.

Yield: 74 %. Elemental analysis: Calcd. for C_7_H_8_O_5_ (172.14): C, 48.84; H, 4.68. Found: C, 49.12; H, 4.51. HPLC-MS (ESI+): 173.22 (100.0%).

^1^H NMR (dmso-d_6_): 3.43 (dd, J= 10.1 Hz, J´=10.1 Hz, 1H, CH), 3.75 (dd, J=7.5 Hz, J´=10.1 Hz, 1H, CH), 4.53 (dd, J=1.52 Hz, J´=5.6 Hz, 1H, CH), 4.95 (ddd, J=0.85 Hz, 1.52 Hz, J´=4.52 Hz, 1H, CH), 5.44 (dd, J=4.52 Hz, 4.80 Hz, 1H, CH), 5.89 (dd, J=1.50 Hz, J=1.50 Hz, 1H, CH), 5.95 (d, J=5.9 Hz, 1H, OH), 7.05 (s(br), 1H, OH).

^13^C NMR (dmso-d_6_): 62.65, 65.6, 78.2, 91.1, 112.5, 172.3, 174.4.

**Preparation of furo[2,3-c]pyran-2-one (4, KAR_2_)**

**Step A:**

To a solution of (4*S*,7*S*)-4,7-dihydroxy-4,5,7,7a-tetrahydro-furo[2,3-c]pyran-2-one (3) (3.44 g, 20.0 mmol) in pyridine (80 mL), ethyl chloroformate (8.68 g, 80.0 mmol) was added dropwise with stirring at 0 °C. The mixture was stirred for a further 60 minutes and evaporated under reduced pressure. The residue was dissolved in ethylacetate (80 mL) and filtered through celite. The filtrate was added dropwise with stirring to hexane (240 mL). The precipitate was filtered off and the filtrate was evaporated under reduced pressure to give carbonic acid (4*S*,7*R*)-7-ethoxycarbonyloxy-2-oxo-4,5,7,7a-tetrahydro-2*H*-furo[2,3-c]pyran-4-yl ester ethyl ester (4.36 g, 69 % yield).

**Step B:**

The (4*S*,7*R*)-7-ethoxycarbonyloxy-2-oxo-4,5,7,7a-tetrahydro-2*H*-furo[2,3-c]pyran-4-yl ester ethyl ester from the previous step (4.35 g, 13.8 mmol) was stirred in a mixture of trimethylamine (7.5 mL) and dichloromethane (45 mL) at room temperature for 5 minutes. The reaction mixture was evaporated under reduced pressure and the residue was dissolved in ethylacetate (20 mL) and filtered through celite. The filtrate was added dropwise with stirring to hexane (180 mL). The precipitate was filtered off and dried in a vacuum dessicator to obtain carbonic acid ethyl ester (*S*)-2-oxo-4,5-dihydro-2H-furo[2,3-c]pyran-4-yl ester (2.21 g, 71 % yield).

**Step C:**

A mixture of carbonic acid ethyl ester (*S*)-2-oxo-4,5-dihydro-2*H*-furo[2,3-c]pyran-4-yl ester (2.00 g, 8.84 mmol) and tetrakistriphenylphosphine palladium (508 mg, 0.44 mmol) in tetrahydrofuran (25 mL) was heated in a sealed tube with stirring under an argon atmosphere for 8 hours. After cooling to room temperature the reaction mixture was filtered through celite, washed with tetrahydrofuran and evaporated under reduced pressure. The crude product was purified by flash chromatography on neutral silica (hexan-ethylacetate, 3:1) to obtain furo[2,3-c]pyran-2-one (4) as a white solid.

Yield: 76 %. Elemental analysis: Calcd. for C_7_H_4_O_3_ (136.10): C, 61.77; H, 2.96. Found: C, 61.52; H, 2.84. HPLC-DAD purity: 99.4 %. HPLC-MS (ESI+, m/z, (rel. int.)): 137.12 (100.0%). GC-MS (EI, 70eV, m/z (rel. int.)): 52.1 (100), 79.0 (72), 136.1 (38)

^1^H NMR (CDCl_3_): 5.44 (d, J=1.02 Hz, 1H, CH), 6.68 (d, J=5.46 Hz, 1H, CH), 7.45 (d, J=5.43, 1H, CH), 7.59 (d, J=1.02, 1H, CH).

^13^C NMR (CDCl_3_): 91.6, 106.2, 132.5, 145.3, 147.3, 152.8, 171.9.

**Preparation of 3-bromo-furo[2,3-c]pyran-2-one (5, KAR-Br)**

A mixture of furo[2,3-c]pyran-2-one (4) (500 mg, 3.67 mmol) and *N*-bromosuccinimide (687 mg, 3.86 mmol) in chloroform (25 mL) was stirred for 2 hours at room temperature. The succinimide was filtered off and the filtrate was evaporated under reduced pressure. The residue was suspended in water (20 ml) and extracted twice with dichloromethane (20 mL). The combined organic phases were washed with brine, dried over sodium sulphate and evaporated under reduced pressure to obtain 3-bromo-furo[2,3-c]pyran-2-one (5) as a yellow solid.

Yield: 93 %. Elemental analysis: Calcd.for C_7_H_3_BrO_3_ (215.00): C, 39.10; H, 1.41. Found: C, 39.48; H, 1.02. HPLC-DAD purity: 98.2 %. HPLC-MS (ESI+): 216.10 (100.0%). GC-MS (EI, 70eV): 50.0 (100), 79.0 (78), 213.9 (23), 215.9 (23).

^1^H NMR (CDCl_3_): 6.68 (d, J=5.37 Hz, 1H, CH), 7.55 (d, J=5.37 Hz, 1H, CH), 7.60 (s, 1H, CH).

^13^C-NMR (CDCl_3_) 64.95, 83.25, 104.26, 131.4, 143.29, 145.72, 150.23.

**Preparation of 3-methyl-furo[2,3-c]pyran-2-one (6, KAR_1_)**

A mixture of 3-bromo-furo[2,3-c]pyran-2-one (5) (63 mg, 0.29 mmol), methylboronic acid (25 mg, 0.44 mmol), palladium diacetate (16 mg, 72.5 µmol), S-Phos (90 mg, 0.218 mmol) and potassium phosphate (70 mg, 0.60 mmol) in toluene (5.0 mL) was heated at 100 °C for 36 hours in a sealed tube under an argon atmosphere. After cooling to room temperature the reaction mixture was filtered through celite and washed with toluene. The filtrate was concentrated under reduced pressure and the residue was suspended in water (20 mL). The suspension was extracted twice with dichloromethane (20 mL). The combined organic phases were washed with brine, dried over sodium sulphate and evaporated under reduced pressure. The crude product was purified by column chromatography on neutral silica (hexan-ethylacetate, 3:1) to obtain 3-methyl-furo[2,3-c]pyran-2-one (6) as a white solid.

Yield: 64 %. Elemental analysis: Calcd. for C_8_H_6_O_3_ (150.13): C, 64.00; H, 4.03. Found: C, 64.12; H, 3.82. HPLC-MS (ESI+): 151.12 (100.0%). GC-MS (EI, 70eV): 121.0 (100), 65.1 (47), 150.1 (39)

^1^H NMR (CDCl_3_): 1.85 (s, 3H, CH_3_), 6.60 (d, J=5.34 Hz, 1H, CH), 7.51 (d, J=5.34 Hz, 1H, CH), 7.58 (s, 1H, CH).

^13^C NMR (CDCl_3_): 7.62, 101.50, 105.20, 128.73, 141.62, 143.54, 150.52, 172.45.
