# Supplementary material for: Quantification of karrikins in smoke water using ultra-high performance liquid chromatography–tandem mass spectrometry
Source: Plant Methods. 2019 Jul 25;15:81. doi: 10.1186/s13007-019-0467-z (PMC6659305; doi:10.1186/s13007-019-0467-z)
Supplement: Supplementary file 2 — Additional file 2. Results of test of stability of karrikin standards in deionised water, pH 7.0. Solutions of KAR1 (black squares) and KAR2 (white diamonds) standards (10−5 M) were incubated for 12 days (short-term treatment; a-b) and 12 weeks (long-term treatment; c-d) at +22 °C (a, c) and +4 °C (b, d). Values are mean ± SD (n = 3). [file 13007_2019_467_MOESM2_ESM.docx]

**

**

**Additional file 2.** Stability of karrikin standards in deionised water, pH 7.0. Solutions of KAR_1_ (black squares) and KAR_2_ (white diamonds) standards (10^-5^ M) were incubated for 12 days (short-term treatment; a-b) and 12 weeks (long-term treatment; c-d) at +22°C (a, c) and +4°C (b, d). Values are means ± SD (n = 3).





**Additional file 3.** Karrikin levels (μmol/l) determined by the standard dilution method. Diluted smoke water (SW) and deionised water (dH_2_O) were spiked with mixtures of KAR_1_, KAR_2_ (0.5 and 5 μmol/l) and KAR-Br (1 μmol/l) then analysed by the presented UHPLC–ESI(+)-MS/MS method. The calculated concentrations of each analyte were compared with the known amounts added to samples – 0.5 μmol/l (a) and 5 μmol/l (b), and the recoveries (%) obtained in each spiking experiment are shown (means ± SD, n = 4).
