# Supplementary material for: Quantification of karrikins in smoke water using ultra-high performance liquid chromatography–tandem mass spectrometry
Source: Plant Methods. 2019 Jul 25;15:81. doi: 10.1186/s13007-019-0467-z (PMC6659305; doi:10.1186/s13007-019-0467-z)
Supplement: Supplementary file 3 — Additional file 3. Karrikin levels (μmol/l) determined by the standard dilution method. Diluted smoke water (SW) and deionised water (dH2O) were spiked with mixtures of KAR1, KAR2 (0.5 and 5 μmol/l) and KAR-Br (1 μmol/l) then analysed by the presented UHPLC–ESI(+)-MS/MS method. The calculated concentrations of each analyte were compared with the known amounts added to samples – 0.5 μmol/l (a) and 5 μmol/l (b), and the recoveries (%) obtained in each spiking experiment are shown (mean ± SD, n = 4). [file 13007_2019_467_MOESM3_ESM.docx]

**Additional file 3.** Karrikin levels (μmol/l) determined by the standard dilution method. Diluted smoke water (SW2) and deionised water (dH_2_O) were spiked with mixtures of KAR_1_, KAR_2_ (0.5 and 5 μmol/l) and KAR-Br (1 μmol/l) then analysed by the presented UHPLC–ESI(+)-MS/MS method. The calculated concentrations of each analyte were compared with the known amounts added to samples – 0.5 μmol/l (a) and 5 μmol/l (b), and the recoveries (%) obtained in each spiking experiment are shown (means ± SD, n = 4).
