# Supplementary material for: Quantification of karrikins in smoke water using ultra-high performance liquid chromatography–tandem mass spectrometry
Source: Plant Methods. 2019 Jul 25;15:81. doi: 10.1186/s13007-019-0467-z (PMC6659305; doi:10.1186/s13007-019-0467-z)
Supplement: Supplementary file 4 — Additional file 4: Fig. S1. The detailed protocol of smoke–water samples. [file 13007_2019_467_MOESM4_ESM.docx]

SW1_1993

SW2_1998

SW3_1994

SW4_1999

SW5_2003

5 kg *Passerina vulgaris* and *Themeda triandra*

5 kg fynbos vegetation

5 kg *Themeda triandra* grass

10 g *Themeda triandra* grass

5 kg fynbos vegetation

The grasses were collected from Ukulinga research farm of the University of KwaZulu-Natal, Pietermaritzburg

Grass was burnt and bubbled through 500 ml distilled water for 45 min

The crude SW solutions were further

sub-diluted viz. 1:100, 1:500; 1:1000 and 1:1500

The SW was stored at 10 ℃ for further use

Quantification of KAR_1_, KAR_2_ through UHPLC-ESI(+)-MS/MS method

The SW was tested against

the germination of Grand Rapids seeds in the dark
