# Supplementary figures and images for: Quantification of karrikins in smoke water using ultra-high performance liquid chromatography–tandem mass spectrometry
Source: Plant Methods. 2019 Jul 25;15:81. doi: 10.1186/s13007-019-0467-z (PMC6659305; doi:10.1186/s13007-019-0467-z)

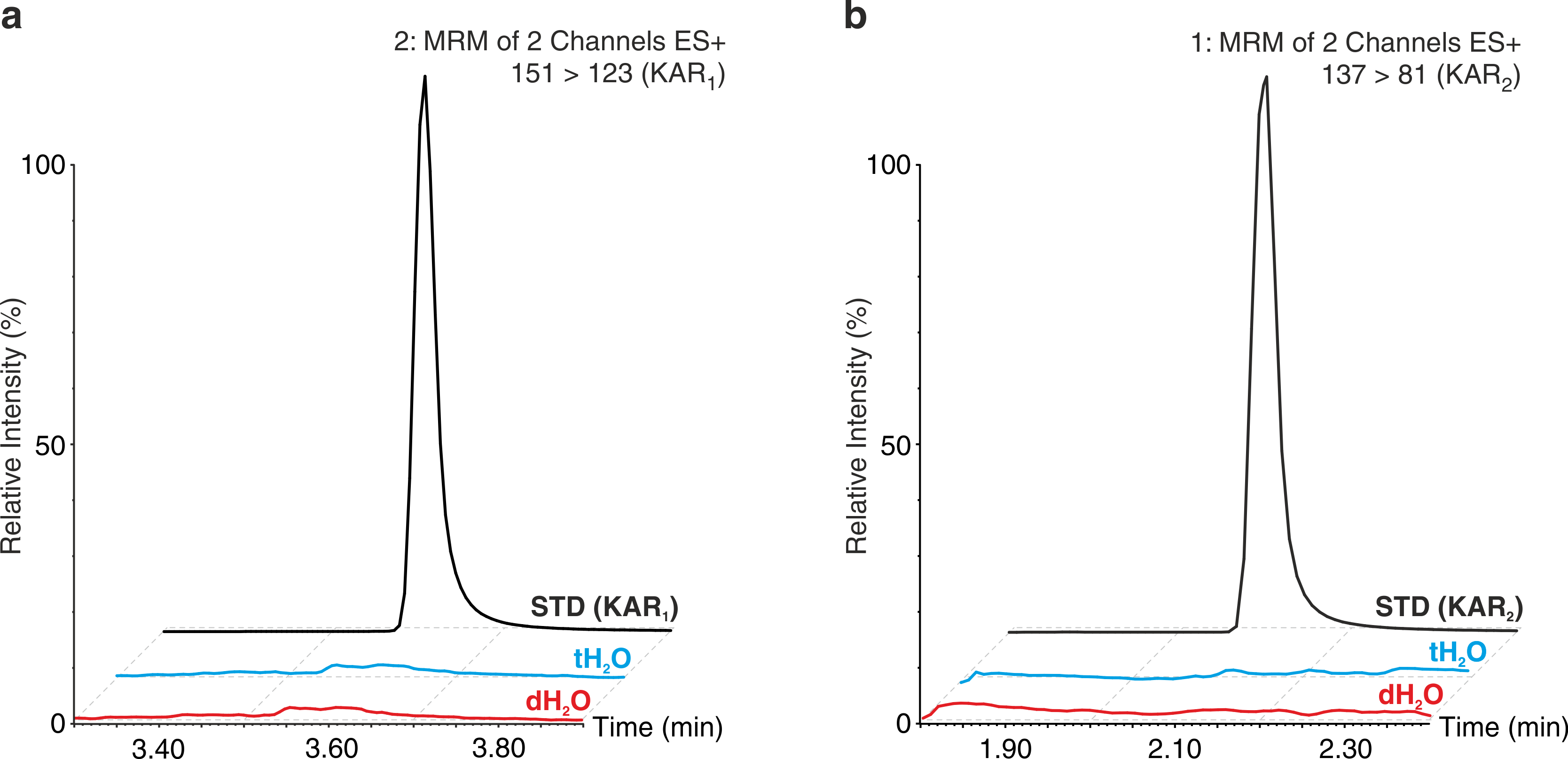

Supplement: Supplementary file 5 — Additional file 5: Fig. S2. Representative MRM chromatograms of pure water samples (tap water injection blue, distilled water injection red, STD injection black). The samples were analysed in triplicate by UHPLC–ESI(+)-MS/MS using the standard addition method. [file 13007_2019_467_MOESM5_ESM.tif]
